# Characteristics of Organellar Genomes and Nuclear Internal Transcribed Spacers in the Tertiary Relict Genus *Dipelta* and Their Phylogenomic Implications

**DOI:** 10.3389/fgene.2020.573226

**Published:** 2020-09-25

**Authors:** Fangfang Peng, Zhe Zhao, Bei Xu, Jie Han, Qian Yang, Yunjing Lei, Bin Tian, Zhan-Lin Liu

**Affiliations:** ^1^Key Laboratory of Resource Biology and Biotechnology in Western China (Ministry of Education), College of Life Sciences, Northwest University, Xi’an, China; ^2^Key Laboratory of Biodiversity Conservation in Southwest China, State Forestry Administration, Southwest Forestry University, Kunming, China

**Keywords:** *Dipelta*, plastomes, mitogenome, intergenic transcribed spacer, divergence, phylogeny

## Abstract

*Dipelta* (Caprifoliaceae) is a Tertiary relict genus endemic to China, comprising three species with horticultural and medicinal values. For the lack of genomic information, interspecific relationships and divergence times in the genus remain unresolved. In the present study, we assembled and characterized the complete plastomes, the partial mitogenomes, and nuclear internal transcribed spacer (ITS) fragments from genome skimming datasets of 14 *Dipelta* individuals. The plastomes were conserved in genomic structure, gene order, and gene content, but with highly variable repeat sequences. Three genes (*rpl23*, *ycf1*, *ycf2*) were examined with positive selection, and nine divergent hotpot regions (*psbL*, *accD*, *rpl23*, *ycf2*, *ycf3*, *rbcL-accD*, *trnI-CAU-ycf2*, *ndhH-rps15*, and *rps18* intron) were potentially valuable for DNA barcodes. Contrasted to the variability in plastome sequences, mitogenomes contained 12 protein-coding genes with limited indels and nucleotide substitutions, and no gene was found under positive selection. Genes in organellar genomes tended to have a similar pattern of codon usage bias, with a preference of A/U ending codons. Phylogenetic trees constructed with plastomes, mitogenomes, and ITS sequences consistently supported that *Dipelta* was monophyletic, and *Dipelta elegans* was sister to the other two taxa. Interspecific divergences were estimated at about 33–37 Ma in the Eocene/Oligocene boundary, suggesting the paleo-endemism of the extant species as “living fossils” of the East Asian Flora. Our study well-exhibited that genome skimming could provide valuable genomic information to elucidate the evolutionary history of the complex group in a cost-efficient way.

## Introduction

**FIGURE 1:**
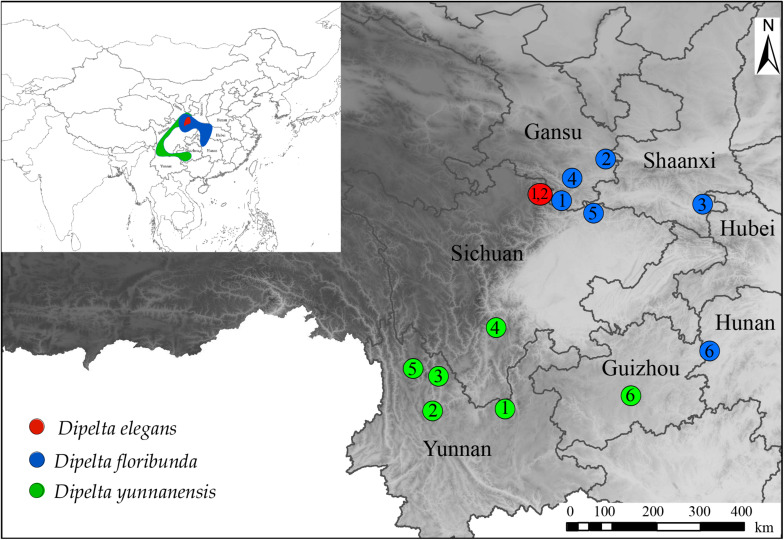
Geographical distribution and sampling locations of *Dipelta* species.

*Dipelta* (Caprifoliaceae), mainly distributed in southwest and central China, is a Tertiary relict woody genus with three extant species, *Dipelta floribunda*, *Dipelta yunnanensis*, and *Dipelta elegans* (Figure 1) (Wu et al., 2011). All species in the genus are of great value in landscape design for their peculiar bell-shaped flowers. *D. yunnanensis* and *D. floribunda* are also used as traditional Chinese medicines to treat pruritus, measles, and damp-heat syndrome (Chen, 2014). Due to the excessive deforestation and continuous deterioration of habitats, *D. yunnanensis* has been listed as a vulnerable species by the International Union for Conservation of Nature. *D. elegans* is also classified as a rare and endangered plant in the China Species Red List for its extremely small population size (Wang and Xie, 2004). Studies on *Dipelta* are generally involved in the phylogenetic analysis of Caprifoliaceae or Dipsacales (Donoghue et al., 2003; Winkworth et al., 2008; Fan et al., 2018; Wang et al., 2020; Xiang et al., 2020). However, interspecific relationships and diversification dating of the genus have not been clearly clarified. Either *D. yunnanensis* or *D. elegans* was possibly a sister to the other two taxa (Liu et al., 2013; Wang et al., 2015). The low resolution of chloroplast fragments even produced a completely polytomous phylogenetic tree of the genus (Liu et al., 2013). According to fossil records in the middle or late Eocene and its historical distribution, *Dipelta* was considered as a paleo-endemic genus in the north temperate flora (Manchester et al., 2009). However, when molecular dating was used to estimate the divergence times among the extant species, controversial conclusions were drawn in previous works. Chloroplast DNA fragments suggested that infrageneric taxa diverged in the Miocene (9.92 Ma) (Wang et al., 2015), indicating the paleo-endemism of the living species. By contrast, Tian et al. (2019) argued that the genus was possibly neoendemic, and speciation occurred in the Pleistocene (0.66 Ma). To elucidate the phylogenetic incongruence and divergence issues, additional genomic data are indispensably needed.

Among the three genomes in plants, plastome is a highly conserved circular DNA molecule with a size of 120–170 kb (Wicke et al., 2011) and has been widely used in studies of species authentication, population genetics, and genetic engineering (Freitas et al., 2018). The internal transcribed spacer (ITS) of nuclear ribosomal DNA (nrDNA) is a well-known marker for phylogenetic works due to its high discrimination power at the species level (Li et al., 2011). Mitogenome has been neglected in plant phylogenetic analysis for its low nucleotide substitution rate, flexible genome structure, and potential gene transfer among intracellular organelles (Zhao et al., 2018; Fonseca and Lohmann, 2020). Recent works revealed that gene regions of mitogenome could also offer valuable phylogenetic information (Bowe et al., 2000; Fonseca and Lohmann, 2020). Furthermore, comparative analysis of plastomes and mitogenomes would provide an illuminating insight into the architectures and evolution of two genomes. Traditionally, PCR-based methods are involved in obtaining DNA sequences. For the lack of genome information, it possibly fails to get genetic data for non-optimal primers or poor quality of DNA. With the rapid advances in high-throughput sequencing technologies, sequence information of three genomes might be gained simultaneously through the genome skimming approach (Straub et al., 2012; Nevill et al., 2020). This low-pass genome sequencing method generates high-copy fractions of total genomic DNA, including the ribosomal DNA, plastome, mitogenome, and other multicopy elements, becoming an efficient tool for phylogenomics and biodiversity researches (Dodsworth, 2015). In the present study, the complete plastomes, partial mitogenomes, and nuclear ITS sequences from 14 individuals of three *Dipelta* species (Figure 1 and Supplementary Table A1) were assembled and annotated through the genome skimming approach. The genomes were compared and subsequently used for phylogenetic analysis. Based on these data, we aim to characterize the variability of organellar genomes, elucidate interspecific phylogenetic relationships, and estimate divergence times of the three *Dipelta* species.

## Materials and Methods

### Sample Collection, Sequencing, and Annotation

Fourteen *Dipelta* individuals (two of *D. elegans*, six of *D. yunnanensis*, and *D. floribunda*, respectively) were collected throughout their natural distribution ranges (Supplementary Table A1 and Figure 1). All voucher specimens were deposited in the Southwest Forestry University Herbarium. Genomic DNA was isolated with the CTAB method from silica gel dried leaves and then sequenced using the Illumina Hiseq 2500 platform in Novogene, Co. Ltd. Raw reads were quality trimmed using the NGS QC Toolkit v2.3.3 (Patel and Jain, 2012) with default cut-off values. The clean reads were mapped to the references using MITObim v1.8 (Hahn et al., 2013) and annotated with DOGMA (Wyman et al., 2004). Genome annotation followed the workflow in our previous work (Peng et al., 2019). The reference sequences were from *D. floribunda* for plastome (MG738670) (Fan et al., 2018) and ITS (FJ745389) (Jacobs et al., 2009). There is no mitogenome information available in Caprifoliaceae. *Chrysanthemum indicum* (MH716014) (Wang et al., 2018) was treated as the reference for mitogenome analysis. All sequences were deposited in GenBank with the accession numbers (Supplementary Table A1).

### Identification of Repeat Sequences

A perl script MISA (Thiel et al., 2003) was used to detect simple sequence repeats (SSRs) loci in the organelle genomes. In addition, the minimum numbers of repeats were set to 10, 5, 4, 3, 3, and 3 for mono-, di-, tri-, tetra-, penta-, and hexa-nucleotides, respectively. The online REPuter program (Kurtz et al., 2001) was employed to identify dispersed and palindromic repeats by setting parameters as (1) Hamming distance of 3; (2) 90% or greater sequence identity; and (3) a minimum repeat size of 30 bp. Additionally, the tandem repeats were screened using the online Tandem Repeats Finder program (Benson, 1999) with the alignment match, mismatch, and indels of two, seven, and seven, respectively. The minimum alignment score and maximum period size were constrained to 80 and 500.

### Comparative Genome and Positively Selected Gene Analysis

Mauve Alignment (Darling et al., 2004) was performed to reveal the rearrangement among plastomes. Sequences were aligned and compared with mVISTA (Frazer et al., 2004) to identify the variation of coding and non-coding regions in plastomes by using one plastome of *D. floribunda* as a reference. The protein-coding genes of plastomes and mitogenomes were used for determining the codon usage pattern. Relative synonymous codon usage (RSCU) was estimated with CodonW (Peden, 1999). In the absence of codon usage bias, the RSCU values would be 1.00. To test the positively selected genes, a maximum likelihood phylogenetic tree was constructed by using RAxML v 7.2.8 (Stamatakis, 2006).

The variations of non-synonymous (dN) and synonymous (dS) nucleotide sites and their ratio (dN/dS or ω) are valuable indicators for the estimation of evolutionary rates and natural selection. We used the codon substitution models to estimate selection pressures, which allowed the ω ratio to vary among sites in all phylogenetic branches. The values of dN, dS, and ω were calculated using the Codeml program in PAML v4.7 (Yang et al., 2005), and the selection pressure was estimated by the site-specific model (seqtype = 1, model = 0, NS sites = 0, 1, 2, 3, 7, 8). Likelihood ratio test (LTR) was performed to estimate the candidate sites of positive selection.

### Phylogenetic Analysis and Divergence Time Estimation

Phylogenetic analysis of the *Dipelta* species was separately conducted using three datasets (the plastomes, 12 mitochondrial genes, and nuclear ITS sequences). Besides *Dipelta*, six Dipsacales species (three Caprifoliaceae and three Adoxaceae species) were selected to construct plastome and ITS phylogenetic trees with an outgroup of *Guizotia abyssinica* (Supplementary Table A1). For the lack of mitogenome information, no other Dipsacales species was included in the present study. The sequences were aligned using MAFFT with the default parameter sets (Katoh and Standley, 2013). The maximum likelihood (ML) analysis was performed using RAxML v 7.2.8 (Stamatakis, 2006) with 1,000 bootstrap replicates. The best-fit model (GTR + G) was determined with Modeltestv3.7 (Posada and Crandall, 1998). We estimated the divergence time of *Dipelta* species using a Yule process speciation prior and GTR substitution model with the uncorrelated relaxed clock in BEAST program (Drummond et al., 2012). We set the fossil calibrations of *Dipelta* and *Kolkwitzia* with mean = 40 Ma, *SD* = 1.0 (Beaulieu et al., 2013; Chen et al., 2018), and the Dipsacales constraint prior was set as a normal distribution, mean = 80 Ma, *SD* = 1.0 (Bell and Donoghue, 2005). The chain length of MCMC was set to 100,000,000 generations, and the sampling frequency was set to 1,000. The effective sample size (>200) was evaluated in Tracer v1.6. The posterior sample of trees was summarized using TreeAnnotator v1.8.0 with the first 10% of the samples discarded as burn-ins (Drummond et al., 2012).

## Results

### Features of ITS and Organellar Genomes

Fourteen *Dipelta* individuals (two for *D. elegans*, six for *D. yunnanensis*, and *D. floribunda*, respectively) were sequenced with genome skimming technology, generating about 2G shallow-coverage genomic data for each individual. The sequencing depths were at least 320, 250, and 2,449 × for plastomes, mitogenome, and ITS, respectively (Supplementary Table A2). Nuclear ITS fragments of *D. yunnanensis* and *D. floribunda* were in similar sizes (613 bp or 61 bp), while *D. elegant* counterparts were longer (623–639 bp) with higher GC contents (Supplementary Table A2). The aligned ITS fragments, containing highly conserved 5.8S rDNA of 160 bp, were totally 639 bp in length with 12 variable sites and 10 parsimony informative sites. Partial mitogenomes of *Dipelta* were also determined in this study, with the sizes of 218,040 bp (*D. floribunda*_GSWX)–242,194 bp (*D. yunnanensis*_YNKM). Twelve intact mitochondrial genes (*atp1*, *atp9*, *mttb*, *atp8*, *cox 3*, *atp 6*, *rpl5*, *rps13*, *ccmb*, *rpl10*, *matR*, and *cob*) were annotated (Supplementary Table A1). The aligned full-length sequences ranged from 351 bp (*rps13*) to 1,533 bp (*atp1*). Among these genes, the *atp8* gene possessed several indels among individuals, showing the highest variable sites (50.6%), but no nucleotide substitution was found in *mttB*, *atp8*, *cox3*, and *ccmB* genes (Supplementary Figure A2). The GC contents of the mitochondrial genes ranged from 37.1% (*atp6*) to 52.0% (*matR*). When the 12 annotated genes were combined, the concatenated sequence was 9,855 bp in length with 199 variable sites (Supplementary Table A1).

The plastome size ranged from 155,114 bp (*D. floribunda*_GSWX) to 155,948 bp (*D. yunnanensis*_SCYX) (Supplementary Figure A1 and Supplementary Table A2). Each plastome exhibited a typical quadripartite structure with a pair of inverted repeat (IR) regions (23,201–23,496 bp) separated by a small single-copy region (SSC, 18,952–19,117 bp) and a large single-copy region (LSC, 83,733–89,975 bp) (Supplementary Table A2). The GC contents in the single-copy (SC) regions of *Dipelta* were lower than IR counterparts, which was ascribed to the high GC contents of rRNA genes in IRs (Dong et al., 2018). There were 128 genes in each plastome, consisting of 82 protein-coding genes, 37 tRNA genes, and eight rRNA genes (Table 1 and Supplementary Table A2). Fifteen genes situated in the IR regions (*rrn4.5*, *rrn5*, *rrn16*, *rrn23*, *trnA-UGC*, *trnI-CAU*, *trnI-GAU*, *trnL-CAA*, *trnN-GUU*, *trnR-ACG*, *trnV-GAC*, *rps7*, *rps12*, *ndhB*, *ycf2*), while the *rpl23* gene located in the junction of the IRb and LSC region. Moreover, 15 intron-containing genes were determined, 12 of which had single intron and three (*rps12*, *rps18*, *ycf3*) had two introns. The *rps12* gene was trans-spliced into three exons, two in IR and one in the LSC region, as reported in other land plants (Howe et al., 2003; Meng et al., 2018).

**TABLE 1:** Genes in the 14 *Dipelta* chloroplast genomes.

**Gene group**	**Gene name**
Ribosomal RNA genes	*rrn4.5^c^*, *rrn5*^c^, *rrn16*^c^, *rrn23*^c^
Transfer RNA genes	*trnA-UGC^ac^*, *trnC-GCA*, *trnD-GUC*, *trnE-UUC*, *trnF-GAA*, *trnG-UCC*, *trnH-GUG*, *trnI-CAU^c^*, *trnI-GAU^ac^*, *trnK-UUU^a^*, *trnL-CAA^c^*, *trnL-UAG*, *trnL-UAA^a^*, *trnM-CAU*, *trnfM-CAU*, *trnN-GUU^c^*, *trnP-UGG*, *trnQ-UUG*, *trnR-UCU*, *trnR-ACG^c^*, *trnS-UGA*, *trnS-GGA*, *trnS-GCU*, *trnT-GGU*, *trnT-UGU*, *trnV-GAC^c^*, *trnV-UAC^a^*, *trnW-CCA*, *trnY-GUA*
Small subunit of ribosome	*rps2*, *rps3*, *rps4*, *rps7*^c^, *rps8*, *rps11*, *rps12*^bc^, *rps14*, *rps15*, *rps16*^a^, *rps18*^b^, *rps19*
Large subunit of ribosome	*rpl2*^a^, *rp114*, *rp116*, *rp120*, *rp122*, *rp123*, *rp132*, *rp133*, *rp136*
DNA-dependent RNA polymerase	*rpoA*, *rpoB*, *rpoC1*^a^, *rpoC2*
Translational initiation factor	*infA*
Subunits of photosystem I	*psaA*, *psaB*, *psaC*, *psaI*, *psaJ*, *ycf3*^b^, *ycf4*
Subunits of photosystem II	*psbA*, *psbB*, *psbC*, *psbD*, *psbE*, *psbF*, *psbH*, *psbI*, *psbJ*, *psbK*, *psbL*, *psbM*, *psbN*, *psbT*
NADH oxidoreductase	*ndhA*^a^, *ndhB*^ac^, *ndhC*, *ndhD*, *ndhE*, *ndhF*, *ndhG*, *ndhH*, *ndhI*, *ndhJ*, *ndhK*
Subunits of cytochrome	*petA*, *petB*, *petD*, *petG*, *petL*, *petN*
Subunits of ATP synthase	*atpA*, *atpB*, *atpE*, *atpF*^a^, *atpH*, *atpI*
Large subunit of Rubisco	*rbcL*
Maturase	*matK*
Envelope membrane protein	*cemA*
Subunit of acetyl-CoA	*accD*
C-type cytochrome synthesis gene	*ccsA*
Protease	*clp*P
Conserved open reading frames	*ycf1*, *ycf2*^ac^

*^a^Gene with one intron.^b^Gene with two introns.^c^Genes located in the inverted repeats.*

### Repetitive Element and SSR Analysis

**FIGURE 2:**
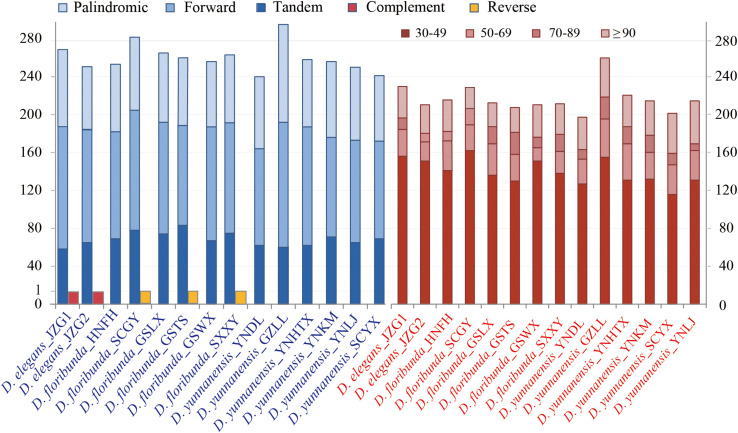
Analysis of repetitive sequences in 14 *Dipelta* chloroplast genomes. The blue histogram on the left indicates the number of five repeat types. The red histogram on the right shows the frequency of the five repeat types by length.

**FIGURE 3:**
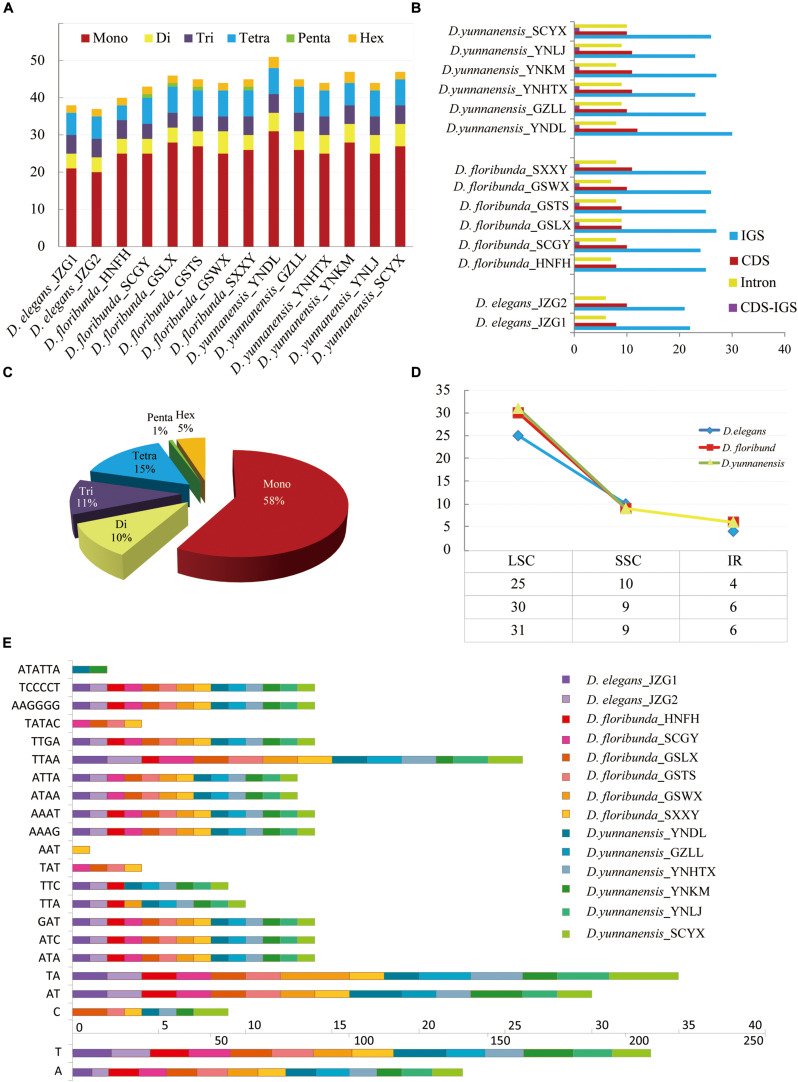
Analysis of simple sequence repeats (SSRs) in 14 plastomes. **(A)** The number of different SSR types detected in each individual. **(B)** Frequency of SSRs in the intergenic regions, protein-coding genes, intron, and CDS-IGS (partly in CDS and partly in IGS). **(C)** Proportion of different SSR types in 14 *Dipelta* individuals. **(D)** Average frequency of SSRs in three species distributed in LSC, IR, and SSC region. **(E)** Type and frequency of each identified SSR.

Repetitive sequences have great values in the study of plant evolution and population genetics. We investigated five category repeats (palindromic, forward, tandem, complement, and reverse repeats) in *Dipelta* species. Plastome analysis showed that *D. floribunda* possessed the greatest number of repeats with the average number of 264, and *D. yunnanensis* had the lowest (256). Forward repetitive sequence was the most frequent, followed by palindromic and tandem repeats in all species (Figure 2). Complement repeats were only found in *D. elegans*, and reverse repeats were only presented in some individuals of *D. floribunda*, both of which were not detected in *D. yunnanensis* (Figure 2). The majority of the repeats were 30–49 bp in length, distributed in intergenic (IGS) or intron regions (Figure 2 and Supplementary Table A4). We also identified six types of perfect SSRs (mono-, di-, tri-, tetra-, penta-, and hexa-nucleotide repeats) in 14 *Dipelta* plastomes. The number of mononucleotides accounted for the largest proportion (58%), while the hexanucleotide was the least (5%), and the pentanucleotide only occurred in *D. floribunda* (Figures 3A,C). Most of SSRs were located in the LSC and IGS regions, which were twice as much as protein-coding regions (Figures 3B,D). Among the repeat units, mononucleotide A/T took up the largest proportion, and all of the dinucleotide repeats were comprised of A and T bases (Figure 3E). Moreover, the ATATTA repeat units were only found in *D. yunnanensis*; the repeat sequences of TATAC, TAT, AAT only appeared in some individuals of *D. floribunda*. In contrast, the mononucleotide repeats of cytosines were not detected in *D. elegans* (Figure 3E).

For mitogenomes, only two forward tandem repeats were found in the *atp9* gene of *D. floribunda*. In addition, penta-nucleotide repeats of (ACTAG)3 were presented in the *matR* gene of all individuals. No SSR was found in the genes of all mitogenomes.

### Comparative Plastome Analysis

**FIGURE 4:**
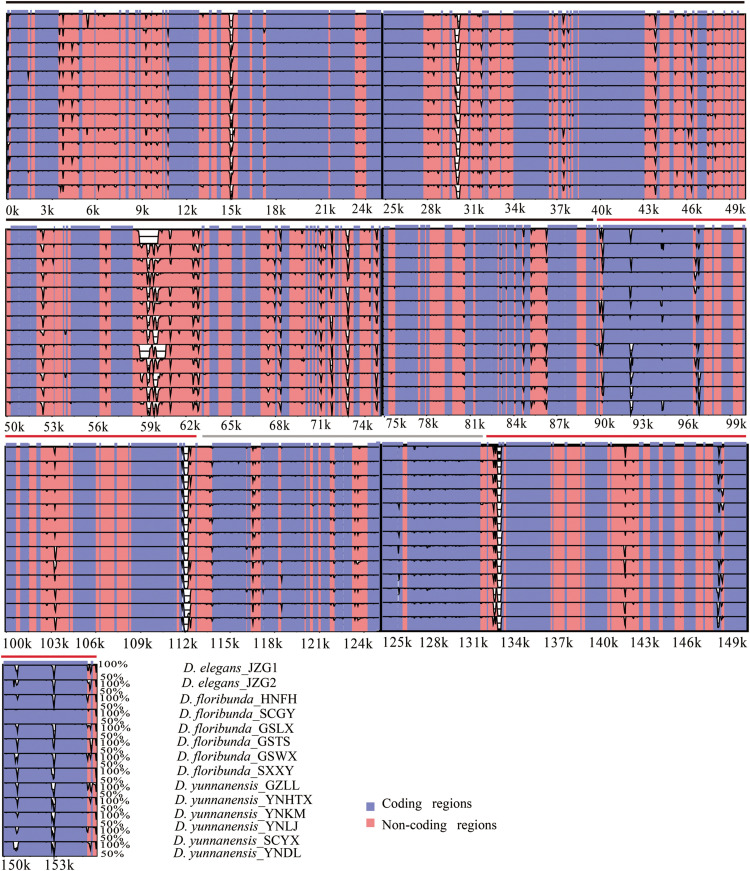
Sequence identity plot of the 14 plastomes in *Dipelta*, with *Dipelta elegans*_JZG1 as a reference. The y-axis indicates% identity ranging from 50 to 100% to the reference. Protein-coding genes and intergenic regions are marked in purple and pink, respectively. The black, gray, and red lines show the LSC, SSC, and IR regions, respectively.

**FIGURE 5:**
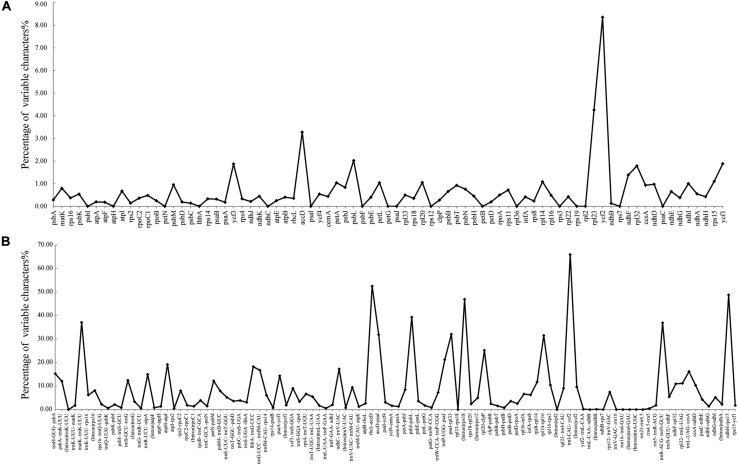
Percentages of variable sites in homologous regions across the 14 *Dipelta* plastomes. **(A)** Protein-coding sequences (CDS). **(B)** The introns and spacers (IGS).

The colinear analysis showed that there was no large-scale structural rearrangements among *Dipelta* plastomes. The overall sequence identities of 14 plastomes were plotted by using the mVISTA program. As expected, high sequence similarity was observed across the plastomes, and the non-coding regions exhibited higher divergence than the coding counterparts (Figure 4). Three genes (*ycf2*, *rpl23*, and *accD*) showed the highest variability with a percentage of variation larger than 0.3%. For the non-coding regions, three intergenic spacers (*trnI-CAU-ycf2*, *rbcL-accD*, and *ndhH-rps15*) and *rps18* intron were more divergent (>45%) than others (Figure 5). These divergence hotspots would provide useful markers for studies at the population level. Interestingly, although some genes (such as *ycf3* and *nadF*) were less variable than the genes mentioned above, their polymorphic sites could still offer valuable information for *Dipelta* species discrimination (Supplementary Table A3).

**FIGURE 6:**
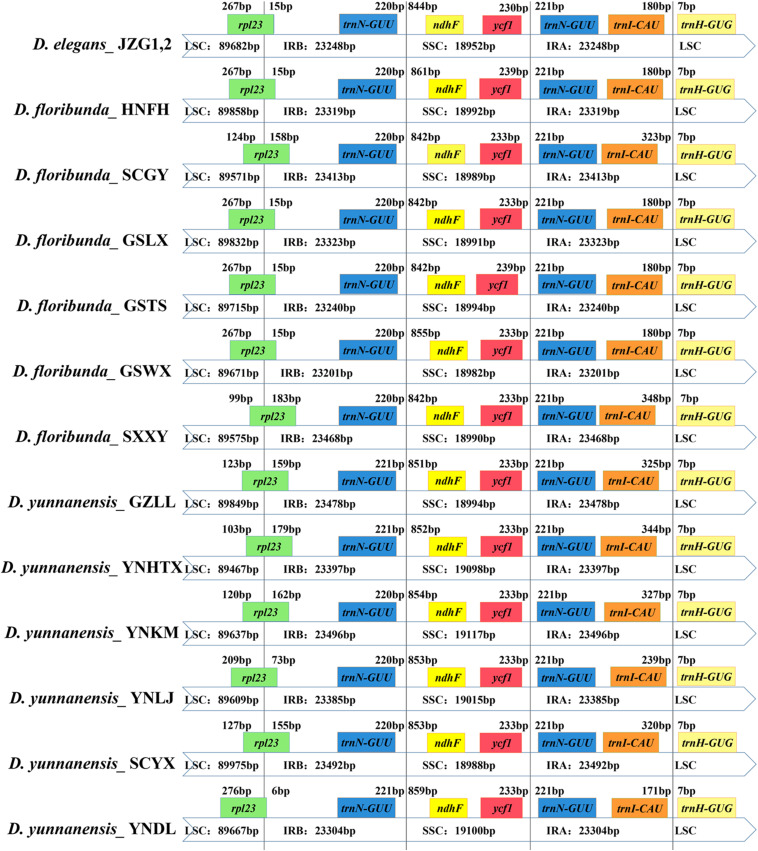
Comparison of the borders of the LSC, SSC, and IR regions in *Dipelta* plastomes.

We compared the SC/IR boundaries and their adjoining genes of 14 plastomes. The junctions of SSC/IR were conserved with a few bases shift. The contraction and expansion of IRs were mainly located in the LSC/IR boundary. The *rpl23* gene straddled the LSC/IRb region and extended to the IR region with 6∼183 bp among different individuals, leading to a distinct shift of the LSC/IRb boundary. The flexibility of LSC/IRa was ascribed to the changeable position of the *trnI-CAU* gene in IRa with 171–348 bp away from the boundaries (Figure 6).

### Codon Usage Pattern and Adaptive Selection Analysis

Here, we analyzed codon usages of the protein-coding genes in the plastomes and mitogenomes from three *Dipelta* species. The number of encoded codons ranged from 25,065 to 25,152 in plastomes and 3,256–3,284 in mitogenomes (Supplementary Table A4). Leucines and cysteines were the most and least abundant amino acids, respectively. UGC for cysteine was the least frequently used codon in both genomes. The codon with the highest frequency was AUU for isoleucine in plastomes, but UUU for phenylalanine in mitogenomes. Codons with A/U at the third nucleotide position were used more frequently than G/C according to the estimation of RSCU values (RSCU > 1) in both genomes (Supplementary Table A4). For instance, 29 of the 30 preferred codons in plastomes (RSCU > 1) were ended with A/U, while only three did so among the 32 less frequently used codons (RSCU < 1). The A/U bias at the third position of codons could also be identified by the AT contents of codons. The mean AT values were 53.5, 61.3, and 68.9% for the first, second, and third positions in plastomes, while the corresponding values were 50.1, 57.4, and 59.7% in mitogenomes, respectively.

The 77 common protein-coding genes in plastomes of *Dipelta* species were used for positive selection analysis (Supplementary Table A5). The genes of *ycf1*, *rpl23*, and *ycf2* potentially had experienced positive selection with 1, 2, and 16 selective sites, respectively (Supplementary Table A5). By contrast, no positive selection site was detected in all 12 mitochondrial genes.

### Phylogenetic Analysis and Molecular Dating

**FIGURE 7:**
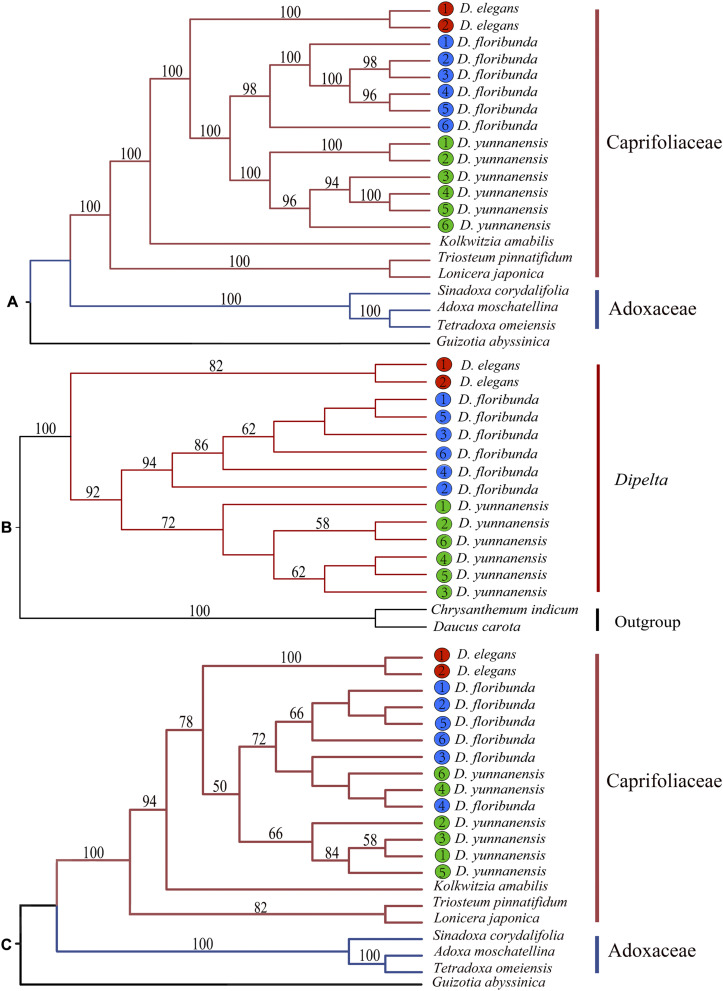
Phylogenetic trees constructed with the complete plastomes **(A)**, 12 mitochondrial combined genes **(B)**, and nuclear ITS sequences **(C)** by using maximum likelihood (ML) analysis. Bootstrap values larger than 50% were labeled above the branches.

Phylogenetic position and interspecific relationships of *Dipelta* were analyzed with the complete plastomes, ITS sequences, and 12 mitochondrial gene sequences, respectively (Supplementary Table A1). Two deep lineages of Caprifoliaceae s.l. and Adoxaceae in Dipsacales were well-supported in the maximum likelihood (ML) tree based on plastomes and ITS sequences as consistently clarified in the previous studies (Fan et al., 2018; Xiang et al., 2020) (Figure 7). Organellar genomic data demonstrated that *Dipelta* was a monophyletic group closely related to *Kolkwitzia*. *D. elegans* was a sister to *D. yunnanensis* and *D. floribunda*. Contrasted to the monophyly of each species in the phylogenetic trees of organellar genomes (Figures 7A,B), two individuals of *D. yunnanensis* were clustered with *D. floribunda* in the ITS tree (Figure 7C), indicating the potential introgression between these two species.

**FIGURE 8:**
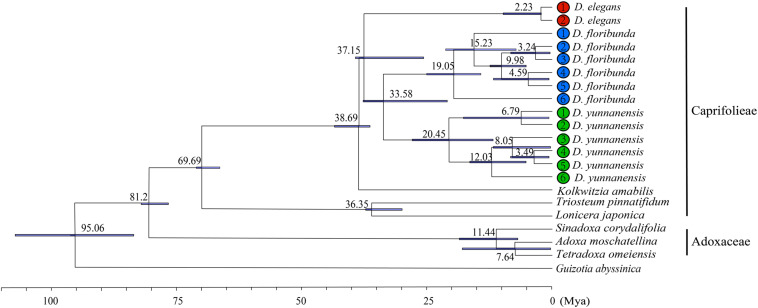
Molecular dating of nine Dipsacales species based on the complete plastomes.

We calculated the molecular divergence time of nine Dipsacales species based on the complete plastomes (Figure 8). The divergence time of the Adoxaceae and Caprifoliaceae s.l. was about 81.2 Ma (95% highest posterior density, 95% HPD = 77.30–82.73 Ma), suggesting the early Campanian divergence of these two families. *Dipelta* split from *Kolkwitzia* in the Eocene, with an estimated age of 38.69 Ma (95% HPD = 36.46–43.56 Ma). The divergence of extant species in *Dipelta* was estimated at about 33–37 Ma (95% HPD = 21.21–39.58 Ma) in the Eocene/Oligocene boundary. The diversification of *Dipelta* was dated in the Miocene, as so did the genera in Adoxaceae (Figure 8).

## Discussion

Genome skimming is an effective approach to produce a large-scale of genomic data for studies in DNA barcoding, biodiversity, and phylogenomics (Straub et al., 2012; Dodsworth, 2015; Nevill et al., 2020). By using this technology, we determined and compared the complete plastomes, partial mitogenomes, and nuclear ITS fragments of 14 individuals from three *Dipelta* species. The 2G raw data generated abundant DNA sequences of three genomes with high sequencing depth. These high-copy fractions of genomic DNA are beneficial to deal with the complex phylogenetic issues unresolved by the traditional DNA sequence fragments (Straub et al., 2012; Trevisan et al., 2019).

### Comparison of Genomic Variation

The plastomes in *Dipelta* were highly conserved in gene numbers, gene orders, and GC contents, with no structural rearrangements. However, the sizes of plastomes were variable within and among species. The contraction and expansion of IRs, as a common evolutionary phenomenon, was regarded as an index of length variation in the angiosperm plastomes (Kim and Lee, 2004). In the 14 *Dipelta* plastomes, the SC/IR boundaries showed similar characteristics with slight shifts in gene positions (Figure 2). The variation of SC/IR borders was considered as a product of the dynamic and random expansion of IRs (Goulding et al., 1996). To evaluate the potential impacts of the junction changes, we analyzed the relationships between the SC/IR boundaries and divergence hotspot regions in 14 plastomes. Contrast to other genes flanking the junctions, the *rpl23* gene spanned the LSC/IR boundary and also had high nucleotide variation, indicating the influence of the shift of the border regions (Figures 5, 6) (Wang and Messing, 2011). The SC regions had a higher sequence divergence than IRs, which might be contributed to copy correction between IR sequences by gene conversion (Khakhlova and Bock, 2006).

Repetitive sequences also played an important role in sequence divergence (Yi et al., 2013). We characterized both complex and simple repeat sequences in the 14 individuals. High diversity in length and nucleotide components was presented in the genus (Figures 2, 3). These abundant repeat sequences provided valuable markers for systematic and population studies (Yi et al., 2013). Interestingly, some repeat sequences tended to exhibit species-specific characters. Component and distribution pattern of SSR were quite similar in *D. floribunda* and *D. yunnanensis*. The complement repeats only appeared in *D. elegans*, and neither the complement repeats nor the reverse repeats were found in *D. yunnanensis*. The reliability of these repeat sequences for species discrimination and phylogenetic analysis is worthy of clarifying in future works.

Among the highly variable genes in *Dipelta*, *ycf2* generally exhibited a high sequence divergence in most of the angiosperms, which was treated as an alternative marker for investigating angiosperm relationships (Huang et al., 2010). The divergence of *rpl23* gene was specific in *Dipelta*. The *accD* gene was considered as a pseudogene in all plastomes of *Dipelta* when the annotation was followed the previous study (Fan et al., 2018). This gene encodes a subunit of acetyl-CoA-carboxylase, which participates in fatty acid synthesis, playing an important role in leaf development (Kode et al., 2005). Its pseudogenization had been reported in several species of Caprifoliaceae (Fan et al., 2018; Liu et al., 2018). Loss or transfer of *accD* was also known in some taxa (Harris et al., 2013; Rousseau-Gueutin et al., 2013). When examining the complete CDS sequences of Caprifoliaceae and Adoxaceae, we found that the *accD* genes in Caprifoliaceae had slight shorter open-reading-frames (ORF) with highly variable 5′-ends and conserved 3′-ends. If being annotated according to plastomes in Adoxaceae, the *accD* gene with complete start and stop codon would be aligned with numerous indels at the 5′-end and tended to be treated as a pseudogene. Therefore, we proposed that the *accD* gene in Caprifoliaceae was functional and annotated in error due to the divergent 5′-ends. However, further studies with more plastome samples are necessarily needed to test its function and divergence in Dipsacales.

Contrasted sharply with the variability of plastomes, mitochondrial genes were considerably conserved. Only 17 polymorphic sites were presented in the 9.9-kb-long sequences. Mitogenome generally contains many repetitive elements and demonstrates frequent gene gain/loss and genomic rearrangements (Christensen, 2013). However, its nucleotide substitution rate in the coding regions is rather low. All 12 mitochondrial genes involved in the study are protein-coding genes, which results in the conserved sequences of the mitogenome. The aligned a*tp9* gene was the most variable fragment with several long indels. High gene diversity of *atp9* was also detected in the natural population of wild carrot (Mandel et al., 2012). However, when blasted in NCBI, besides annotated *atp9 per se*, the sequence could also match the intergenic spacer in the reference mitogenome with high expected values. Therefore, further works are needed to test whether the *atp9* gene is functional.

### Codon Bias and Adaptive Selection

The codon usage pattern is essential for cellular function and is considered as an important factor for species divergence and molecular evolution (Gu et al., 2018). In plastomes and mitogenomes of *Dipelta*, each individual showed a similar codon usage pattern according to the RSCU values (Supplementary Table A4). However, preferred codons differentially existed between the two cytoplasmic genomes. As an example, UAA for the stop codon was preferred in plastomes, while a bias to UGA was favored in mitogenomes. Preferred codon usage bias was found in ACC for threonine and GCC for alanine in mitogenomes, both of which were less used in plastomes. The difference in favored codon usage pattern was closely related to the origin and evolutionary history of two organellar genomes (Xu et al., 2015). In *Triticum aestivum*, neutral mutation and translation coupled factors played a critical role in shaping the codon usage pattern of mitogenome. For plastomes, selected constrain was potentially a major factor leading to the codon usage bias (Zhang et al., 2007). Despite the same tendency of A/U at the third codon position, the AT contents of the third position in mitogenomic codons were illuminatingly lower than those in plastomes (59.7 vs. 68.9%). The difference of codon GC usage between two genomes could be ascribed to several factors, such as GC contents of genes, mutation bias, natural selection, transcription levels, etc. (Xu et al., 2015; Khan et al., 2018).

Positive selection analysis was performed for protein-coding genes of plastomes and mitogenomes. Only three chloroplast genes, *rpl23*, *ycf1*, and *ycf2*, were identified under positive selection pressure. The *rpl23* gene, encoding the large subunit ribosomal protein 23, was also one of the divergent hotspot regions. The *ycf1* and *ycf2* genes, the two largest chloroplast genes encoding the envelope membrane protein translocon in the chloroplast (Kikuchi et al., 2013), are essential for cell survival in plants (Drescher et al., 2000). They generally evolved at rapid mutation rates (Zhou et al., 2019) and were reported as selected genes in several species of Caprifoliaceae (Fan et al., 2018; Liu et al., 2018). Three allopatric *Dipelta* species possess heterogeneous habitats (Tian et al., 2019). These positively selected genes may have played significant roles for *Dipelta* species to adapt to the diverse environments.

### Phylogenetic Relationships and Divergence Age Estimation

Interspecific relationships of the small endemic genus of *Dipelta* were still unclear (Liu et al., 2013; Wang et al., 2015). In this study, we successfully applied the plastome, mitogenome, and nuclear ITS sequences to elucidate the phylogenetic issues of *Dipelta*. Our result suggested that *Dipelta* was monophyletic and closely related to *Kolkwitzia*, as identified in systematic works of Caprifoliaceae (Jacobs et al., 2010; Wang et al., 2015; Landrein et al., 2017). Within the genus of *Dipelta*, all data supported that *D. elegans* was a sister to the other two species. This was also supported by SSR and AFLP data (Liu et al., 2013), but inconsistent with chloroplast fragment sequences, in which *D. yunnanensis* was the earliest divergent species (Wang et al., 2015). The incongruence was mainly ascribed to the limited sampling number (one individual per species) and chloroplast data (nine fragments) in the latter study. *Dipelta elegans* is the most restricted species distributed only in the north Qinghai-Tibet Plateau (QTP) of China. *D. elegans* has unique morphological traits, i.e., the largest epicalyx and the smallest nectary bulge with the entire calyx (Landrein and Farjon, 2020). It also harbors many species-specific nucleotide components shared with *Kolkwitzia amabilis*, which might be ancestral rather than recent derived or convergent (Supplementary Table A3). Liu and colleagues proposed that *D. elegans* retreated to the current distribution area during the glacial period as several species in QTP (Xu et al., 2010). The widespread allopatric distribution of *D. yunnanensis* and *D. floribunda* was an outcome of population expansion in the opposite direction during the period of Pleistocene (Tian et al., 2019). In the nuclear ITS tree, two individuals of *D. yunnanensis* were grouped with *D. floribunda* (Figure 7C). These individuals of *D. yunnanensis* are located in the marginal region of its natural range adjacent to *D. floribunda*. The incongruence between nuclear and cytoplasmic phylogenetic trees could be explained as interspecific introgression in the past. Introgressive hybridization is commonly found in their sympatric zone (Liu et al., 2013) and possibly occurred before the last interglacial period (Tian et al., 2019).

The divergence times about Caprifoliaceae species have been estimated by using the complete chloroplast genomes with fossil calibrations (Fan et al., 2018; Wang et al., 2020). We uncovered the split of the Adoxaceae and Caprifoliaceae s.l. at about 81.2 Ma at the Cretaceous/Tertiary boundary, similar to the previous study (Fan et al., 2018) but younger than that announced by Wang et al. (2020). The dating bias was partly due to the difference in samples/data and fossil constraints (Wang et al., 2020). *Dipelta* originated in the Eocene (36.46–43.56 Ma) (Chen et al., 2018) and diversified in the Eocene/Oligocene boundary (33–37 Ma) (Figure 8), supporting the hypothesis of pre-Oligocene intergeneric divergence and post-Eocene infrageneric diversification in Caprifoliaceae (Wang et al., 2020). The divergence age of *Dipelta* species was much older than those recently reported (Wang et al., 2015; Tian et al., 2019). Interestingly, *Dipteronia*, another Tertiary relict woody genus with two allopatric species, has similar geographical distribution pattern to *Dipelta*: one species locates in Central China, like *D. floribunda*; the other was restricted to Southwest China, as *D. yunnanensis*. The interspecific divergence within *Dipteronia* was dated back to the early Miocene (31.19 Ma) (Bai et al., 2017) or the early Eocene (52.7 Ma) (Feng et al., 2019). The unexpectedly identical divergent ages indicated that the diversification of these two Tertiary relict genera was potentially traced to a common history event, the global climate transit from the greenhouse to the icehouse conditions in the Paleogene (Katz et al., 2008). The divergence between the south and north species in *Dipelta* and *Dipteronia* potentially resulted from parallel evolution, driven by geographical isolation and natural selection of the heterogeneous environments. Surviving the climate oscillations in the late Tertiary and Quaternary, they become the living fossils in the East Asian flora.

## Data Availability Statement

The datasets presented in this study can be found in online repositories. The names of the repository/repositories and accession number(s) can be found in the article/ Supplementary Material.

## Author Contributions

Z-LL conceptualized the study, wrote, reviewed, and edited the manuscript. FP and QY were responsible for the software. ZZ, BX, and JH were in charge of validation. FP and YL were responsible for the formal analysis. BT was in charge of the resources. FP prepared and wrote the original draft. All authors have read and agreed to the published version of the manuscript.

## Conflict of Interest

The authors declare that the research was conducted in the absence of any commercial or financial relationships that could be construed as a potential conflict of interest.

## Supplementary Material

SUPPLEMENTARY TABLE A1 List of species and their GenBank accession numbers in the study.

SUPPLEMENTARY TABLE A2 Features of plastomes, mitogenomes and ITS fragments in *Dipelta* species.

SUPPLEMENTARY TABLE A3 Selected genes in plastome and mitogenome with potential applications in species discrimination.

SUPPLEMENTARY TABLE A4 Codon usage and relative synonymous codon usage (RSCU) value for protein-coding genes in *Dipelta* cytoplasmic genomes.

SUPPLEMENTARY TABLE A5 Likelihood ratio test (LRT) of the variable ω ratio under different models.
